# Sacituzumab govitecan in triple-negative breast cancer: from bench to bedside, and back

**DOI:** 10.3389/fimmu.2024.1447280

**Published:** 2024-08-15

**Authors:** Valentina Rossi, Alessandra Turati, Antonio Rosato, Debora Carpanese

**Affiliations:** ^1^ Immunology and Molecular Oncology Unit, Veneto Institute of Oncology (IOV)-IRCCS, Padova, Italy; ^2^ Department of Surgery, Oncology and Gastroenterology, University of Padova, Padova, Italy

**Keywords:** triple negative breast cancer (TNBC), sacituzumab govitecan (IMMU-132), antibody-drug conjugate (ADC), Trop-2, immunotherapy, target therapeutics, metastatic TNBC

## Abstract

Triple-negative breast cancer (TNBC) represents a major therapeutic challenge due to its heterogeneous and aggressive phenotype, and limited target-specific treatment options. The trophoblast cell surface antigen (Trop-2), a transmembrane glycoprotein overexpressed in various cancers, has emerged as a promising target for TNBC. Sacituzumab govitecan (SG), an antibody-drug conjugate (ADC) that targets Trop-2, has recently entered treatment algorithms for advanced and metastatic TNBC, independently from Trop-2 expression status, with manageable toxicity. Despite the impressive results, questions remain unsolved regarding its efficacy, safety profile, and Trop-2 biological role in cancer. Currently, Trop-2 cannot be designated as a predictive biomarker in SG treatment, albeit its expression correlates with disease outcome, yet its levels are not uniform across all TNBCs. Additionally, data regarding Trop-2 expression variations in primary and metastatic sites, and its interplay with other biomarkers are still ambiguous but mandatory in light of future applications of SG in other indications and settings. This poses the questions of a careful evaluation of the efficacy and toxicity profile of SG in such early stages of disease, and in personalized and combinatorial strategies. Research and clinical data are mandatory to address SG drawbacks and minimize its benefits, to realize its full potential as therapeutic agent in different epithelial tumors.

## Introduction

Breast cancer (BC) is one of the leading causes of female mortality (1), where triple-negative breast cancer (TNBC) subtype represents 15%-25% of all cases. This malignancy is characterized by high invasiveness, metastatic potential to distant sites, such as bone, lung, liver, and brain (2), leading to increased mortality rates, proneness to relapse (3), and short survival after the onset of metastatic disease (4). The aggressive tumor behavior and limited treatment options highlight the urgency to identify novel prognostic biomarkers and cancer-specific therapeutic targets to improve patient outcomes. Trophoblast cell surface antigen 2 (Trop-2) is a 35 kDa transmembrane calcium signal transducer glycoprotein, overexpressed in various epithelial tumors, representing a valuable target for managing malignancies with limited treatment options, such as TNBC (5). Antibody-drug conjugates (ADCs) are an emerging category of antineoplastic treatments that combine the selectivity of monoclonal antibodies (mAbs) with the potency of cytotoxic drugs (6). This approach has been successfully exploited, leading to the accelerated approval of sacituzumab govitecan (SG, IMMU-132) for the treatment of locally advanced or metastatic TNBC (mTNBC), suggesting a new era in TNBC treatment. However, questions about the efficacy and safety profile of SG are arising, as well as the still uncovered multiple biological interplays with different molecules and the predictive or prognostic value of its target Trop-2 in cancer. Here, in an attempt to shedding light on these unmet topics, we discuss recent data about TNBC treatment with SG from trials and clinical practice.

## Triple-negative breast cancer: treatment options and new promising possibilities

The aggressive tumor behavior and the lack of expression of estrogen (ER) and progesterone (PR) receptors, along with the absence of human epidermal growth factor receptor 2 (HER2) overexpression (7), lead to the deficiency of targeted therapeutic solutions for TNBCs. Consequently, treatment options rely on surgical excision, radiation, and nonspecific chemotherapy, with limited efficacy and short progression-free survival (PFS), especially in patients who have previously received therapies in the metastatic setting (8). Despite these efforts, over 50% of TNBC patients experience relapse within 3-5 years, with a median overall survival (OS) of only 10.2 months (9). Moreover, all TNBC subtypes are directly associated with epithelial-to-mesenchymal transition (EMT), which plays a role in the development of drug resistance and further complicating treatment (10). In recent years, several promising therapeutic algorithms have emerged for TNBC. BRCA-targeted therapies such as olaparib (11) and talazoparib (12), exploit DNA repair deficiencies in *BRCA*-mutated TNBC. Immune checkpoint inhibitors (atezolizumab, pembrolizumab) have improved outcomes in programmed death-ligand 1 (PDL1)-positive mTNBC when combined with chemotherapy (13). Novel therapies targeting HER2-low expression (trastuzumab deruxtecan) show promise (14), while anti-androgen therapies (bicalutamide, enzalutamide) are explored in androgen receptor (AR)-positive TNBC (15). Inhibitors of the PI3K/AKT/mTOR pathway (alpelisib, everolimus) (16) and other targeted treatments like MEK inhibitors (trametinib) (17) are also alternative strategies still under investigations. However, such options are exploitable only for specific TNBC subgroups, and thus the identification of attractive targets in the therapeutic hunt for TNBCs is still an open challenge, together with the design and validation of effective ADCs. These included ladiratuzumab vedotin and patritumab deruxtecan that target LIV-1 and HER3, respectively. Despite the promising objective response rate (ORR), such treatments demonstrated suboptimal safety profiles (18).

## Trop-2 as a therapeutic target: state of art

**Figure 1:**
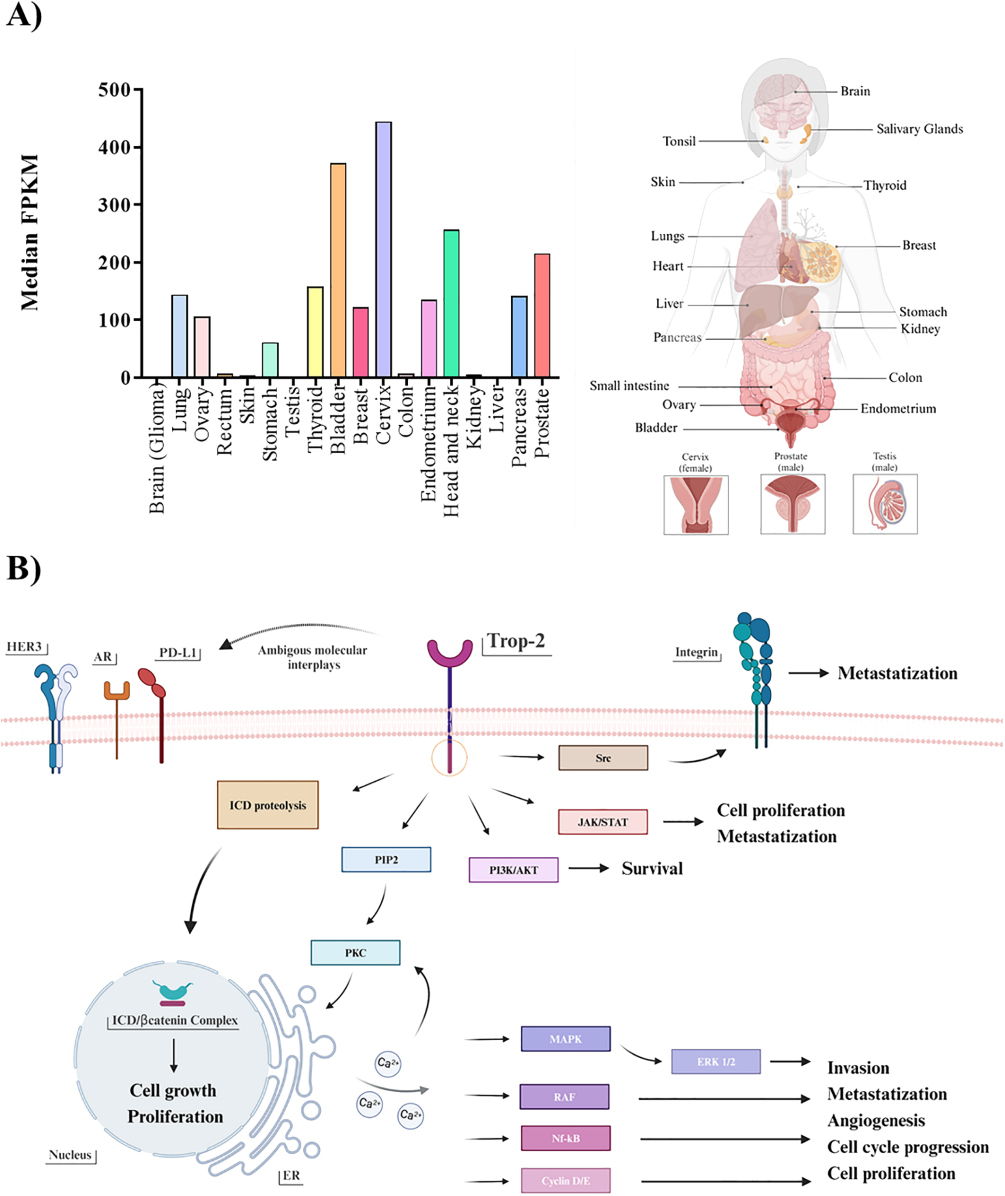
**(A)** Trop-2 mRNA profile. Boxplots summarizing the mRNA quantification of Trop-2 in pathological malignancies (left). Values represent the median number Fragments Per Kilobase of exon per Million reads (FPKM), generated by The Cancer Genome Atlas (TCGA). Values from brain, testis and liver ranging up to 10 median FPKM result not visible. All described tissues are indicated in the correspondent body district (right). **(B)** Trop-2 downstream pathways. Schematic resume of Trop-2 molecular downstream mechanisms triggered in cancerous setting: ERK1/2MAPK pathway emerges in all main tumorigenic, proliferative and metastatic processes, alongside with the other PIP2/PKC-induced calcium-regulated downstream pathways (RAF/Nf-kB/Cyclin D/E) (24). JAK/STAT and PI3K/AKT were described to be implicated in survival, proliferation and metastatic processes (25), as for the Src mediated intervention on the integrin-fibronectin axis (26). In addition, regulated proteolysis of Trop-2 was found to drive proliferative and self-renewal signaling via β-catenin (27). Possible ambiguous interplays occurring between Trop-2 and other plausible receptor already cited in the text are also illustrated.

Among promising therapeutic target candidates, Trop-2 has recently emerged as one of the most promising. It was firstly identified as widely expressed in various healthy epithelial and mucosal tissues (19–21) where it plays an essential role in stem cell proliferation, embryonic development and placental tissue formation (22, 23). In pathological context, it recently arouses curiosity due to the anomalous overexpression of both protein and mRNA in various solid cancers (**Figure 1A**) (28–31), where it is associated with aggressive tumor characteristics, such as enhanced growth, invasion, and metastasis (32–36). Moreover, in occurrence of conventional treatments failure, enhanced Trop-2 expression levels have been associated with therapeutic success, paving the way to the exploitation of a novel class of therapeutics (37). Indeed, the correlation of Trop-2 expression with disease and/or clinical outcome in multiple tumor types, and the lower expression in normal adult tissues compared to several pathological counterpart (38–41), underscores the potential benefit of targeting Trop-2 to fill an unmet need in cancer treatment. Trop-2 is overexpressed across all BCs, especially in aggressive subtypes such as TNBC and HR+/HER2-, where it resulted a strong predictor of lymph node involvement, distant metastasis, and poor OS (42). Of note, its oncogenic potential was highlighted in TNBC, where it was linked to oncogenic metabolism elements, therapeutic resistance, poorer OS even at early stages (43, 44), leading to the approval of ADCs for metastatic malignancy even though only as third line treatment (45, 46). Moreover, its consistent high expression on circulating-tumor-cells, makes it a valuable potential marker for EMT and initiation of metastatic processes (47). Remarkably, as recently highlighted by the ASCENT trial results, TNBC patients belonging to the two highest Trop-2-expressing quartiles demonstrated an improvement in both PFS and OS, exhibiting better response rates to the treatment compared to those with low Trop-2 expression (48). In addition, data from clinical trials have reported response rates to SG not exceeding the 35%, augmenting the perplexity about how the absence or very low levels of Trop-2 expression may have contributed to therapy failures and enhanced side effects (49). In fact, it is crucial to consider the possibility of enhanced risk of side effects, due to its ubiquitous nature in widely represented normal tissues, as squamous epithelial and mucosa (50, 51).

On the other hand, it is pivotal to remark that the recruitment strategy of TNBC patients in ADC-based trials is most likely due to the limited therapeutic options available for those who have experienced progression after at least two prior therapies, rather than to a particularly high rate of Trop-2 expression. As matter of fact, as showed in a recent work by Dum et al., among a total subset of 18,563 different tissue tumor microarrays examined (n=2,139 for BC), TNBC does not rank among the top cancers with high Trop-2 expression, with only 54.4% of cases being strongly positive for the receptor (52). These findings may suggest that ranking tumor types by Trop-2 expression frequency and intensity may help identify a more precise approach to select patients for successful therapeutic outcome. Alongside, a further advancement in proper personalized treatment approaches and positive therapeutic rebound may be represented by following up Trop-2 level throughout the whole therapeutic regimen, starting from the early stages of the disease. Indeed, rigorous monitoring should be advocated not only concerning HR phenotype of metastases, but also focusing on Trop-2 expression fluctuations, related to metabolic and transcriptional plasticity induced by EMT (53). On this line, the establishment of a standardized detection methodology with diagnostic utility may aid in the comprehensive analysis of the receptor, essential for efficient tumor stratification and expression status check-up (54). Due to the complexity of Trop-2 induced downstream network (**Figure 1B**), attention was called to the differential role of Trop-2 in a tumor-type dependent manner, as recent studies indicated the promotion of tumorigenesis in concomitance with the loss of Trop-2, depending on the cell type and context (32, 55). Indeed, identifying reliable associated biomarkers is also essential to aid patient selection and prediction of therapeutic responses. In this regard, observations were reported in literature concerning in both prostate cancer and TNBC, where Trop-2 and AR showed interconnection in their expression (56, 57). Furthermore, in head-and-neck squamous cell carcinoma, the reduction of Trop-2 expression resulted in sensitivity to anti-HER3 antibodies (58) while in non-small cells lung cancer it was observed that Trop-2 overexpression was linked to the primary resistance to PD-L1 blockade, suggesting an interconnection existing between the two receptors, and thus recommending the administration of combinatorial therapy solely after a Trop-2-based selection (59). This emphasizes the pleiotropic nature of Trop-2 biology and interaction patterns, warranting further exploration of its tumorigenic downstream pathways and the consequential influence of these ambiguous molecular interplays in its potential biomarker/therapeutic target role, uncovering the reason behind suboptimal treatment outcomes and therapy failures.

## Sacituzumab govitecan: a new era for immunotherapy of TNBC (with future challenges)

**Figure 2:**
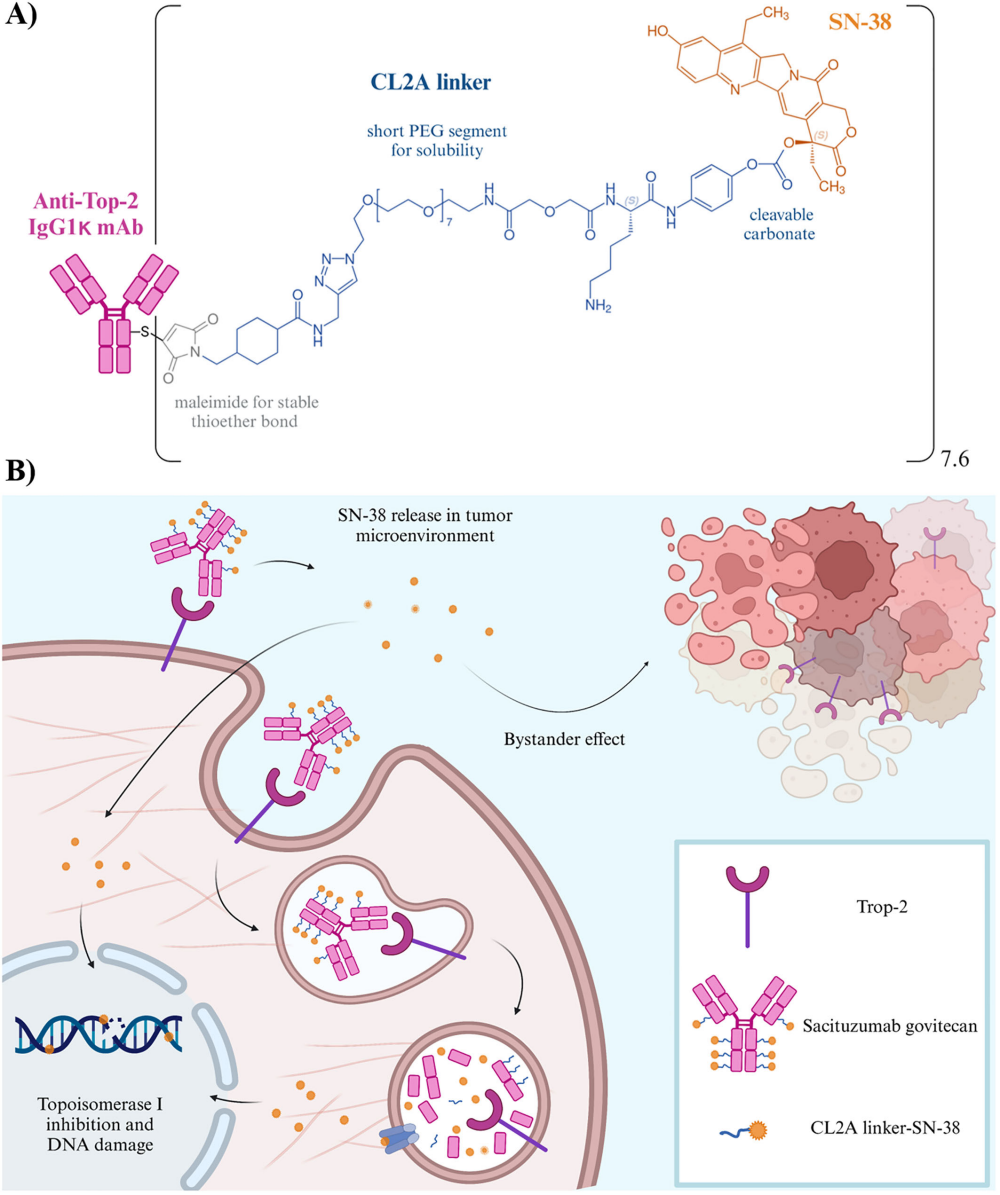
**(A)** Structure of SG. Schematic representation of the chemical structure of the linkage of SN-38 (shown in orange) to the hRS7 antibody (pink) via the CL2A-linker (blue). Specifically, the CL2A linker binds to the 20^th^ position of SN-38, stabilizing the lactone ring and forming a pH-sensitive carbonate bond. The presence of a short polyethylene glycol (PEG) segment enhances the water solubility of such SN-38 conjugate. Moreover, the maleimide inserted at the end of the linker enables a stable thioether bond with sulfhydryl moieties formed after mild reduction of the hRS7 mAb. The average DAR is 7.6 (61). **(B)** Mechanism of action of SG. i) The ADC recognizes and binds to Trop-2 on the tumor cell, being then be internalized; ii) the payload undergoes intracellular trafficking and enters the lysosomes following antibody catabolism and hydrolysis of the linker, the payload is released and induces apoptotic cell death. Neighboring cancer cells are affected by the bystander effect (64, 65), due to the release of SN-38 from the target cell or within the extracellular space, thus contributing to an amplified anti-tumor effect (61, 66). The unconjugated hRS7 mAb showed some effector function *in vitro* (*i.e*. antibody-dependent cellular cytoxicity), which resulted mitigated in the ADC due to mAb reduction for conjugation purposes (67, 68).

SG is a third-generation ADC that exploits the humanized anti-Trop-2 mAb hRS7 (IgG1κ) to deliver SN-38, the active metabolite of the topoisomerase I inhibitor irinotecan. Both the hydrophobicity and high potency of SN-38 preclude its direct use in the clinic, with patients experiencing grade 3/4 diarrhea (60). Conversely, SG delivers higher levels of SN-38 with an improved safety and tolerability profile (61–63). This is the result of SG unique design, in which SN38 is linked to the mAb through the hydrolizable pH-sensitive linker CL2A, allowing the delivery of up to 8 moieties per single molecule (**Figure 2A**) (61). Overall, SG represents a breakthrough in TNBC treatment, as a paradigm shift and challenge in ADC design, since the majority of ADCs delivers ultra-toxic payloads (IC_50_ values of pM ranges and often narrow therapeutic index). The lower potency of SN38 (IC_50_ of nM) enables a high drug to antibody ratio (DAR), and the administration of the highest clinical dosing regimen of all Food and Drug Administration (FDA)-approved ADCs: 10 mg/kg administered on days 1 and 8 of a 21-day cycle (69), avoiding side effects usually associated with other ADCs, such as interstitial lung disease or pneumonitis (70). The putative mechanism of action of SG is summarized in **Figure 2B**. IMMU-132-01 was the first phase I/II multicenter, single-arm, basket study to evaluate the single-agent activity, safety and tolerability of SG. The study enrolled patients with different advanced epithelial cancers, refractory to/relapsed after at least one standard line of chemotherapy in the metastatic setting. Subjects with active brain metastasis or under systemic corticosteroids for more than 2 weeks before enrolment were not eligible. Notably, the 69 patients with mTNBC were heavily pretreated, with a median of 5 (range 1-12) prior lines of therapies. Twenty-one subjects (30%) achieved an ORR, and the median duration of response (DoR) at the time was 8.9 months (95% CI, 6.1-11.3). Both *BRCA* germline status and Trop-2 expression were evaluated (60% primary tumors and 40% miscellaneous metastases), with 7/43 patients being *BRCA1* mutated and 42/48 subjects having moderate-high Trop-2 staining. Consistent with preclinical findings showing greater antitumor effects of SG in mice bearing tumors with high Trop-2 expression (62, 67, 68), a positive trend was observed in PFS for patients with 2+ or 3+ Trop-2. Conversely, subjects bearing weak or negative Trop-2 levels obtained only stable disease as best response (71). In 2019, efficacy results were furtherly confirmed in a larger cohort of mTNBC, with an ORR 33.3% (95% CI, 24.6-43.1), a median time to response (TTR) of 2.0 months (1.6-13.5 range), and a median DoR of 7.7 months (95% CI, 4.9-10.8). A clinical benefit was observed in 49 patients (45.4%). For survival endpoints, the PFS was 5.5 months (95% CI, 4.1-6.3) and OS reached 13 months (95% CI, 11.2-13.7), with a manageable toxicity profile (49). Efficacy and safety in mTNBC were further evaluated in the phase III confirmatory ASCENT trial, in comparison to the treatment of physician’s choice (TPC). The primary endpoint was PFS in patients without brain metastases, while secondary endpoints included PFS in the intention-to-treat population, OS in both populations, ORR, DoR, quality of life (QoL), and safety. SG outperformed TPC in terms of PFS (5.6 months versus 1.7 months; HR 0.41, 95% CI 0.32–0.52) and OS (12.1 months vs 6.7 months; HR 0.48, 95% CI 0.38–0.59) in the population without brain metastases, with an ORR of 31% versus 4% in the full population (72). The trial was halted early in March 2020 due to compelling evidence of efficacy, granting accelerated approval by the FDA and the European Medicine Agency (EMA) for patients with unresectable locally advanced or mTNBC who received two or more prior lines of therapy, including at least one for metastatic disease. Parallelly, SG was also approved for advanced HR-positive, HER2-negative BCs that had already received endocrine-based therapy and at least two additional systemic therapies in the metastatic setting (TROPiCS-02) (73). In April 2021, FDA granted accelerated approval for the treatment of advanced urothelial cancer after platinum and PD-1/PD-L1-directed checkpoint inhibitors (TROPHY-U-01) (74). Updated results from the ASCENT trial confirmed the previous data, with SG improving outcomes over TPC despite Trop-2 expression level. However, a trend in Trop-2 subgroups is noticeable: in the lower quartiles of Trop-2, the improvement of clinical outcomes appeared to be less pronounced, with PFS of 2.7 months (HR 0.58, 95% CI 1.4-5.7) and OS of 8.7 (HR 0.74, 95% CI 6.9-12.9). These data, even if explorative, suggest that also Trop-2-low mTNBCs may benefit from SG, as occurred for trastuzumab deruxtecan in HER2-low BCs (44). Adverse events (AEs) observed were consistent with those reported in previous trials (46). The most common registered toxicities of any grade included neutropenia (the prevalent reason for treatment interruption), nausea, diarrhea, fatigue, anemia, and vomiting. The most frequent grade ≥ 3 AEs for SG were neutropenia (52% vs 34%), febrile neutropenia (6% vs 3%), diarrhea (12% vs 1%), leukopenia (11% vs 6%), anemia (9% vs 6%), nausea and abdominal pain (both 3% vs ~1%), primarily associated with SN-38 and manageable with supportive care. Unlike other ADCs, SG did not show an increased risk of interstitial lung disease or cardiovascular toxicity, with patients reporting a meaningful improvement in QoL (75). Interestingly, no correlation between AEs ad Trop-2 status has emerged (48). Overall, these data supported the potential of SG to mark a substantial advancement toward pretreated mTNBC. Nevertheless, many questions are still unresolved. Since SG, as other ADCs, is gaining increasing interest as first-line agent or in the adjuvant setting (**Table 1**), its long-term efficacy, safety and reversibility of side effects are becoming of paramount importance, as the response rates may drastically vary based on indications and settings. The geriatric population (age ≥ 65 years) showed a tendency to experience higher rates of SG discontinuation due to AEs. However, such difference in the ASCENT study was not statistically significant, probably because this population is a minority (SG and TCP together, 19%) (48, 72). Trials concerning other tumor histotypes in which geriatrics were much more represented (74, 76) reported increased discontinuation rates for SG, highlighting the necessity to further investigate such subpopulation in TNBCs. Real-world studies and pharmacovigilance safety databases will contribute to a clear comprehension of AEs in elderly and other subgroups (*i.e.*, ethnical minorities, patients with ECOG-PS ≥ 2, and/or with active brain metastases, *etc.*). Indeed, the few available studies, while confirming the efficacy of SG, also pointed out the complexity of such populations and the necessity of a thorough evaluation and understanding of some AEs such as alopecia and pneumonitis (77–79). As discussed above, Trop-2 expression evolution in cancer is still unclear, and samples analyzed in clinical trials were a miscellaneous of primary and various metastatic sites. Its overexpression may be detected at very early-stages of disease, even during tumor formation, opening new possibility to a prompt therapeutic intervention for TNBC, and posing questions about a universal method for Trop-2 detection. Despite being well-established and validated, the immunohistochemical analysis reported in SG clinical trials differed in terms of antibody selection for Trop-2, ranging from polyclonal (80) to murine mAb (81), mining interpretation and comparison of such data. Additionally, some chemotherapies, *i.e.* irinotecan itself, may alter Trop-2 expression (82), which seems also to be directly involved in promoting drug resistance (59, 82, 83). In this connection, the relapse of cancer patients responding to SG raises the question of resistance against the cytotoxic payload and/or the mAb. The mechanisms underlying resistance to ADCs are likely to be complex. While data concerning resistance to the unconjugated mAb counterparts are available for other ADCs (84–86), knowledge regarding SG is more than preliminary. On one hand, topoisomerase inhibitors are known to induce multidrug resistance (87), while on the other, it was recently demonstrated that distinct acquired resistance mutations involving both Trop-2 and topoisomerase I emerged within different metastatic lesions of a SG-treated patient (88). The management of SG-derived AEs poses the question whether these events may depend on SN-38 itself or Trop-2-bound ADC in off-target tissues. ADCs may bind and then be internalized in healthy tissues which express low levels of the target antigen, even though the majority of AEs is likely attributable to off-target events or release of the cytotoxic payload in the bloodstream. Moreover, toxicity in normal cells may be related to ADC uptake mediated by Fcγ receptors, with cells expressing high levels of mannose receptors such as in myeloid, endothelial and hepatic cells tissues, displaying higher abilities to interact with a relatively high proportion of galactosylated glycans on the Fc domain of the mAb, favoring off-target toxicities (89, 90).

**Table 1:** Active/recruiting trials testing SG in BCs (updated 2024.06.03).

NCT Number/Trial name	Study Phase	N° of patients	Patient cohort	Treatment arms	Primary outcome	Status
NCT04230109 NeoSTAR	Phase II	260	Localized TNBC	SG in neoadjuvant setting. 1°cohort: SG monotherapy for 4 cycles, then TPC. 2°cohort: SG + pembrolizumab for 4 cycles. Future planned arms will include SG +/- pembrolizumab and/or TPC for patients with HR+ BC and IBC	pCR	Recruiting
NCT03424005 Morpheus-panBC	Phase I/II	242	Locally advanced and mTNBC immunotherapy-naive	Multiple immunotherapy-based combinations (total of 12 cohorts), with one cohort: atezolizumab + SG	ORR, n° of patients with AEs	Recruiting
NCT05113966	Phase II	30	Locally advanced and mTNBC who received at least 2 prior treatments, 1 in the metastatic setting	Single arm: SG + trilaciclib	PFS (up to 24 months)	Active, not recruiting
NCT03971409 InCITe	Phase II	150	Locally advanced and mTNBC	Multiple avelumab-based combinations (total of 6 cohorts), with one cohort: avelumab + SG	BORR	Recruiting
NCT05382299 ASCENT-03	Phase III	540	Previously untreated PD-L1 negative locally advanced and mTNBC/PD-L1 positive locally advanced TNBC and mTNBC previously treated with an ICI	1°cohort: SG monotherapy 2°cohort: TPC (paclitaxel, nab-paclitaxel, or gemcitabine + carboplatin)	PFS (up to approximately 22 months)	Recruiting
NCT05382286 ASCENT-04	Phase III	440	PD-L1 positive locally advanced and mTNBC	1°cohort: SG + pembrolizumab 2°cohort: pemrbrolizumab + TPC (paclitaxel, nab-paclitaxel, or capecitabine)	PFS	Recruiting
NCT05633654 ASCENT-05	Phase III	1514	TNBC that completed surgery	1°cohort: SG + pembrolizumab in adjuvant setting (8 cycles) 2°cohort: TPC (paclitaxel, nab-paclitaxel, or gemcitabine + carboplatin)	iDFS (up to 60 months)	Recruiting
NCT05840211 ASCENT-07	Phase III	654	HR+/HER2- locally advanced or mBC who received endocrine therapy	1°cohort: SG 2°cohort: TPC (paclitaxel, nab-paclitaxel, or capecitabine)	PFS (up to 29 months)	Recruiting
NCT04468061 (Saci-IO)	Phase II	110	PD-L1 negative mTNBC	1°cohort: SG 2°cohort: SG + pembrolizumab	PFS (3 years)	Recruiting
NCT04039230	Phase I/II	75	mTNBC	Single arm: SG + talazoparib	Dose limiting toxicity (2 years)	Recruiting
NCT05552001 ISIdE	Phase III	96	Locally advanced and mTNBC who received at least 1 prior treatment	Single arm: SG	ORR	Recruiting
NCT04434040 ASPRIA	Phase II	40	Localized TNBC	Single arm: SG + atezolizumab (6 cycles) in adjuvant setting	Rate of undetectable circulating tumor cfDNA - 6 cycles	Active, not recruiting
NCT04647916	Phase II	44	HER2- mBC and brain metastasis	Single arm: SG in adjuvant setting (2 years)	Intracranial ORR (time frame: up to 2 years)	Recruiting
NCT04958785 ELEVATE TNBC	Phase II	92	Locally advanced and mTNBC who received 1 prior line of therapy in advanced setting	Safety run-in 1°cohort: magrolimab + nab-paclitaxel or paclitaxel Safety run-in 2°cohort: magrolimab + SG Phase II 1°cohort arm A: magrolimab + nab-paclitaxel or paclitaxel Phase II 1°cohort arm B: nab-paclitaxel or paclitaxel Phase II 2°cohort: magrolimab + SG	Safety run-in cohort: DLT, AEs. Phase II 1°cohort: PFS. Phase II 2°cohort: ORR	Active, not recruiting
NCT05143229 ASSET	Phase I	18	Locally recurrent or HER2- mBC	Dose level 1: alpelisib 250 mg OA daily + SG 8 mg/kg IV on days 1 and 8 of each 21-day cycle Dose level 2: alpelisib 250 mg OA daily + SG 10 mg/kg IV on days 1 and 8 of each 21-day cycle Dose level 3: alpelisib 300 mg OA daily + SG 10 mg/kg IV on days 1 and 8 of each 21-day cycle	Recommended phase II dose of alpelisib + SG	Recruiting
NCT04448886	Phase II	110	HR+/HER2- mBC	1°cohort: SG 2°cohort: SG + pembrolizumab	PFS (time frame: 3 years)	Active, not recruiting
NCT04595565 SASCIA	Phase III	1332	HER2- localized BC	1°cohort: SG in adjuvant setting (8 cycles) 2°cohort: TPC (capecitabine or platinum-based)	iDFS	Recruiting
NCT06328387	Phase I/II	120	Advanced BC	1°cohort: SG 2°cohort: HCQ + SG 3°cohort: T-DXd 4°cohort: HCQ + T-DXd	DLT, AEs, ORR (time frame: 2 years)	Recruiting
NCT06100874 SATEEN	Phase II	40	mHER2+ mBC	Single arm: SG + Trastuzumab (or Biosimilar) IV or SC (with hyaluronidase)	ORR (time frame: 3 years)	Recruiting

AEs, adverse events; BORR, best overall response rate; cfDNA, circulating free DNA; DLT, dose limiting toxicity; HCQ, hydroxychloroquine; IBC, inflammatory breast cancer; ICI, immune checkpoint inhibitor; iDFS, invasive disease free survival; mBC, metastatic breast cancer; mTNBC, metastatic triple-negative breast cancer; OA, oral administration; IV, intravenous administration; ORR, objective response rate; pCR, pathological complete response; PFS, progression free survival; SC, subcutaneous administration; SG, sacituzumab govitecan; T-DXd, trastuzumab deruxtecan; TPC, treatment of physician’s choice.

## Conclusions and outlook

SG has emerged as a promising option in TNBC due to its high efficacy and safety profile and unique mechanism of action. On the other hand, it is crucial to highlight that the road to success is paved with challenges, especially for entering new treatment algorithms in different contexts and settings. Indeed, “old” challenges are still present, which are basically related to patient selection and biomarker assessment. As research and clinical experience with SG continue to grow, a precise analysis leading back to bench aimed at addressing its limitations and enhancing its efficacy profile, will pave solid bases for exploiting its full potential in the management not only of TNBC and other malignancies, but also to promote the improvement of other Trop-2 targeting strategies.
